# Malignant transformation of an intraparenchymal hemangioma in the cervical spinal cord of a German shepherd dog

**DOI:** 10.1111/jvim.17190

**Published:** 2024-09-11

**Authors:** Courtney P. Korff, Sophie Nelissen, Amy B. Todd‐Donato, Andrew D. Miller, Emma Davies

**Affiliations:** ^1^ Department of Clinical Sciences, Section of Neurology Cornell University College of Veterinary Medicine Ithaca New York USA; ^2^ Department of Population Medicine and Diagnostic Sciences, Section of Anatomic Pathology Cornell University College of Veterinary Medicine Ithaca New York USA; ^3^ Department of Clinical Sciences, Section of Diagnostic Imaging Cornell University College of Veterinary Medicine Ithaca New York USA

**Keywords:** canine, hemangioma, hemangiosarcoma, intraparenchymal, primary spinal cord neoplasia

## Abstract

An 8‐year‐old female spayed German shepherd dog was presented for evaluation of a 1‐week history of right thoracic limb monoparesis. Magnetic resonance imaging (MRI) identified an intraparenchymal, T2 hypointense and T1 isointense, strongly heterogeneously contrast‐enhancing mass with moderate internal susceptibility artifact on T2* images at the level of the cranial extent of the C5 vertebral body. Euthanasia was elected after a rapid neurologic decline in the 24 hours after MRI. Necropsy and histopathology identified an intraparenchymal hemangiosarcoma arising from a hemangioma in the cervical spinal cord, with no evidence of neoplastic disease in any other examined organs. The spectrum of vasoproliferative disorders in the central nervous system in veterinary species has been codified recently, but hemangiosarcoma is considered metastatic to the central nervous system. Herein we describe the clinical, imaging, and histologic findings in a dog with a novel primary location of hemangiosarcoma in the cervical spinal cord.

**Abbreviations**
CNS: central nervous system
CSF: cerebrospinal fluid
MRI: magnetic resonance imaging
PPAR: peroxisome proliferator‐activated receptor

## 1 INTRODUCTION

Hemangiosarcoma (HSA) is a malignant neoplasm of vascular endothelial origin.^1^ Originally thought to arise secondary to malignant transformation of vascular endothelial cells, recent evidence suggests a pluripotent bone marrow progenitor cell origin in which abnormal cell maturation results in vascular tumor formation.^1^, ^2^ Mutations in normal tumor suppressor genes as well as dysfunctional angiogenesis also may contribute to development.^1^, ^3^, ^4^, ^5^, ^6^ Consequently, the pathogenesis of HSA, like other neoplasms, is likely multifactorial. Hemangiosarcoma is characteristically a highly aggressive neoplasm typified by early metastasis both to regional and distant organs, frequently including the lungs, liver, and peritoneum and carries a poor prognosis.^1^, ^7^, ^8^ Although any breed is at risk, this tumor type is more prevalent in German shepherd dogs, Labrador retrievers, and golden retrievers.^7^, ^8^, ^9^

With regard to the central nervous system (CNS), vascular proliferations can be broadly divided into benign and malignant groups. Benign vascular malformations in the CNS are dominated by hemangiomas, although a wide range of tumor types have been reported.^10^ Hemangiosarcoma can be deleterious through both local invasion and metastatic disease.^11^ It is the most common tumor to metastasize to the brain and an estimated 14% of dogs with visceral HSA have intracranial metastasis.^12^, ^13^

Although HSA is the most common malignant primary splenic and cardiac neoplasm in the dog,^1^ primary HSA of the CNS is exceedingly uncommon in domestic animals and not reported in dogs.^14^ It may lead to myelopathy through spinal cord compression, predominantly associated with vertebral neoplasia, which may be primary or metastatic, although primary epidural HSA occasionally has been described.^11^, ^15^, ^16^, ^17^, ^18^ Intraparenchymal HSA thus far has been associated only with secondary metastasis to the spinal cord.^11^, ^19^

We describe the clinical presentation, magnetic resonance imaging (MRI) appearance, and histopathologic findings of a primary intraparenchymal HSA arising from a hemangioma in the cervical spinal cord of a German shepherd dog.

## 2 CASE PRESENTATION

An 8‐year‐old spayed female German Shepherd dog was evaluated at the Cornell University College of Veterinary Medicine Hospital for Animals for dragging of the right thoracic limb after playing roughly with other dogs 1 week prior. The patient's clinical signs had remained static despite grapiprant (Galliprant, Elanco, Greenfield, Indiana, USA) administration at an unknown dose by the referring clinician. Physical examination identified pain on extension of the stifle and coxofemoral joints bilaterally without identifiable instability. Neurologic examination disclosed a marked monoparesis of the right thoracic limb with absent withdrawal reflex and proprioceptive placing or hopping in this limb. Proprioceptive testing and spinal reflexes were normal in the remaining limbs. Cranial nerve examination and cutaneous trunci reflexes were normal. Pain was elicited with lateral flexion of the cervical spine to either side. The neurolocalization was right‐lateralized C6‐T2 myelopathy or brachial plexus.

SNAP 4D× testing for heartworm, *Borrelia burgdorferi, Ehrlichia*, and *Anaplasma* was negative. Venous blood gas identified mild respiratory alkalosis (pH, 7.397; reference range, 7.32‐7.38; pCO2, 36.6 mmHg; reference range, 38‐46), mildly increased lactate concentration (2.77 mmol/L; reference range, 0‐2), mild hypernatremia (153 mEq/L; reference range, 145‐151), and mild hypokalemia (3.89 mEq/L; reference range, 3.9‐5.1). Complete blood count showed mildly increased hematocrit (60%; reference range, 41‐58) and increased total protein concentration (9.1 g/dL; reference range, 5.9‐7.8). A biochemistry panel showed mild hypernatremia (151 mEq/L; reference range, 143‐150), mild hypokalemia (3.6 mEq/L; reference range, 4.1‐5.4), mildly increased total protein concentration (7.5 g/dL; reference range, 5.5‐7.2), mild hypoglycemia (63 mg/dL; reference range, 68‐104), and mildly increased creatine kinase activity (438 U/L; reference range, 61‐314). No abnormality was detected on thoracic radiographs. Cervical radiographs showed in‐situ mineralization of the C6‐7 intervertebral disk.

**FIGURE 1:**
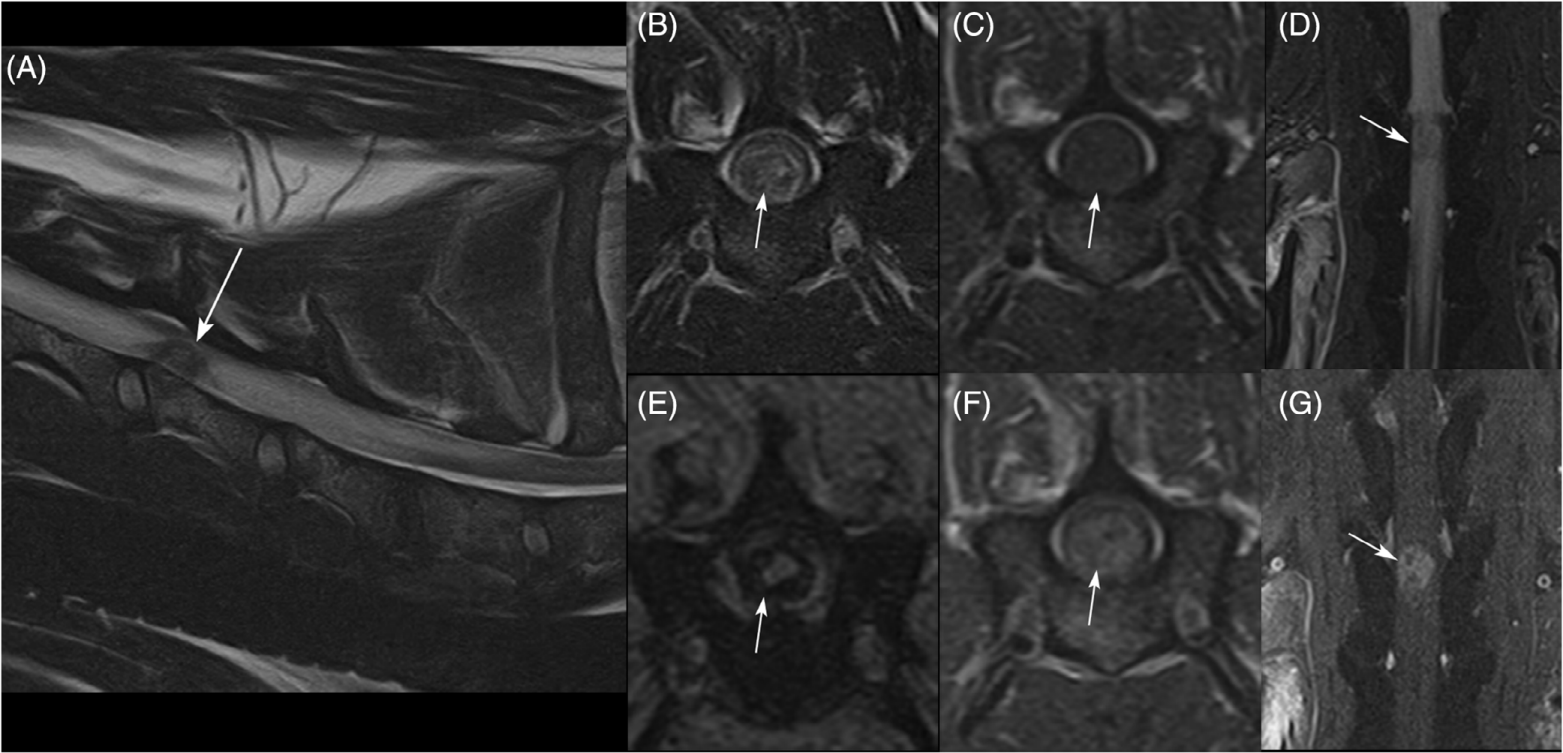
Magnetic resonance images of the suspected focal intraparenchymal spinal cord mass over the cranial body of the C5 vertebra. The white arrows indicate the lesion. The lesion was hypointense to muscle on sagittal (A) and transverse (B) T2w sequences, isointense on transverse T1w (C) sequences with strong heterogenous contrast enhancement on transverse T1 + C (F) and dorsal T1 + C fat saturation sequences (G). The dorsal STIR sequence (D) showed moderate hyperintensity in the subscapularis muscles bilaterally and the right supraspinatus muscles and marked swelling and hyperintensity of the spinal cord cranial and caudal to the lesion. The T2* (E) sequence showed a moderate amount of intraparenchymal mixed susceptibility artifact.

The patient was anesthetized for cervical MRI using a 1.5T scanner (Vantage Atlas, Canon Medical Systems USA, Inc., Tustin, California, USA). Magnetic resonance imaging identified a medium‐sized intraparenchymal mass within the ventral aspect of the spinal cord parenchyma causing marked, focal enlargement of the spinal cord at the level of the cranial extent of the C5 vertebral body, with compression of the unaffected spinal cord parenchyma at the level of the mass, which assumed a semilunar shape dorsal to the mass (Figure 1). The mass was round, T2 hypointense (relative to muscle), T1 isointense (relative to muscle), and strongly heterogeneously contrast‐enhancing. The mass also displayed a moderate amount of internal susceptibility artifact on T2*‐weighted images. Regionally, the spinal parenchyma cranial and caudal to the mass (spanning C2‐C7) was swollen, markedly T2‐hyperintense, T1 iso‐ to hypointense, and did not exhibit contrast enhancement. These findings were most consistent with neoplasia, with hemorrhagic glioma or metastatic HSA prioritized. Lumbar cerebrospinal fluid (CSF) collection was performed and analysis was consistent with albuminocytologic dissociation (total nucleated cell count, 1 cells/uL; (reference range, 0‐5); red blood cell, 82 cell/uL; (reference range, 0); and total protein concentration, 148 mg/dL; (reference range, 0‐35)).

Abdominal ultrasound examination was performed after MRI and identified moderate to severe, diffuse, nodular splenopathy with innumerable, small to medium sized, poorly marginated, round, moderately hypoechoic nodules. Both benign and neoplastic causes were considered. Ultrasound‐guided fine needle aspiration was performed and cytology results were consistent with normal splenic elements with no evidence of neoplastic transformation.

Palliative management using 0.15 mg/kg dexamethasone‐SP IV q24 was initiated. However, the patient's neurologic status declined over the next 24 hours to plegia of the thoracic limbs with absent superficial but intact deep pain sensation bilaterally and mild paresis of the pelvic limbs (considered more severe on the right side). Euthanasia was elected at that time, and the dog was submitted for necropsy.

## 3 GROSS DESCRIPTION AND HISTOLOGIC DESCRIPTION

**FIGURE 2:**
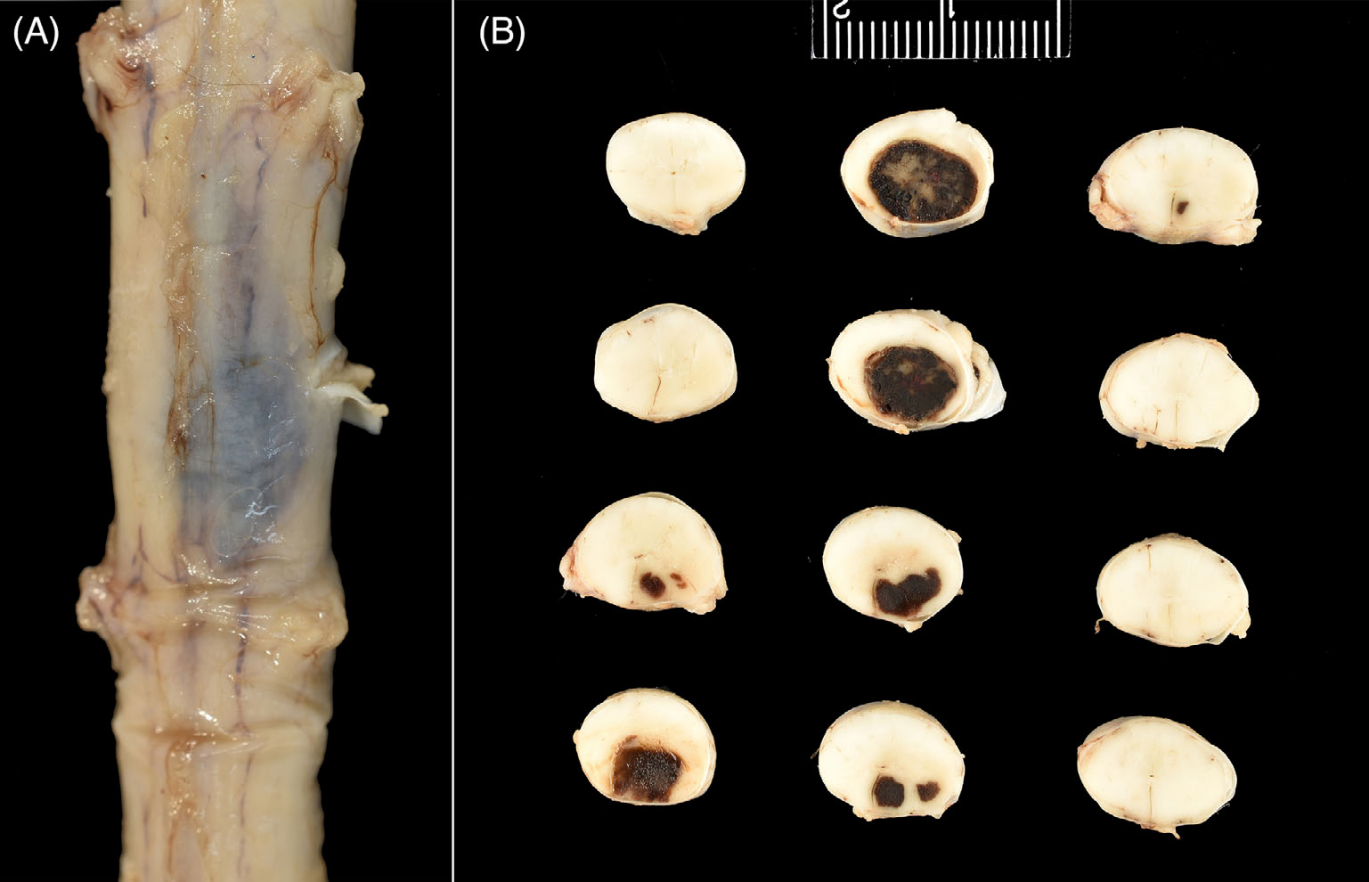
Gross image of the cervical mass. The mass is ovoid, relatively well‐delineated, dark brown to red, and dry on section. As evidenced in both the ventral view of the spinal cord (left) and the serial sections (right), hemorrhagic tendrils extend cranially and caudally to the mass.

**FIGURE 3:**
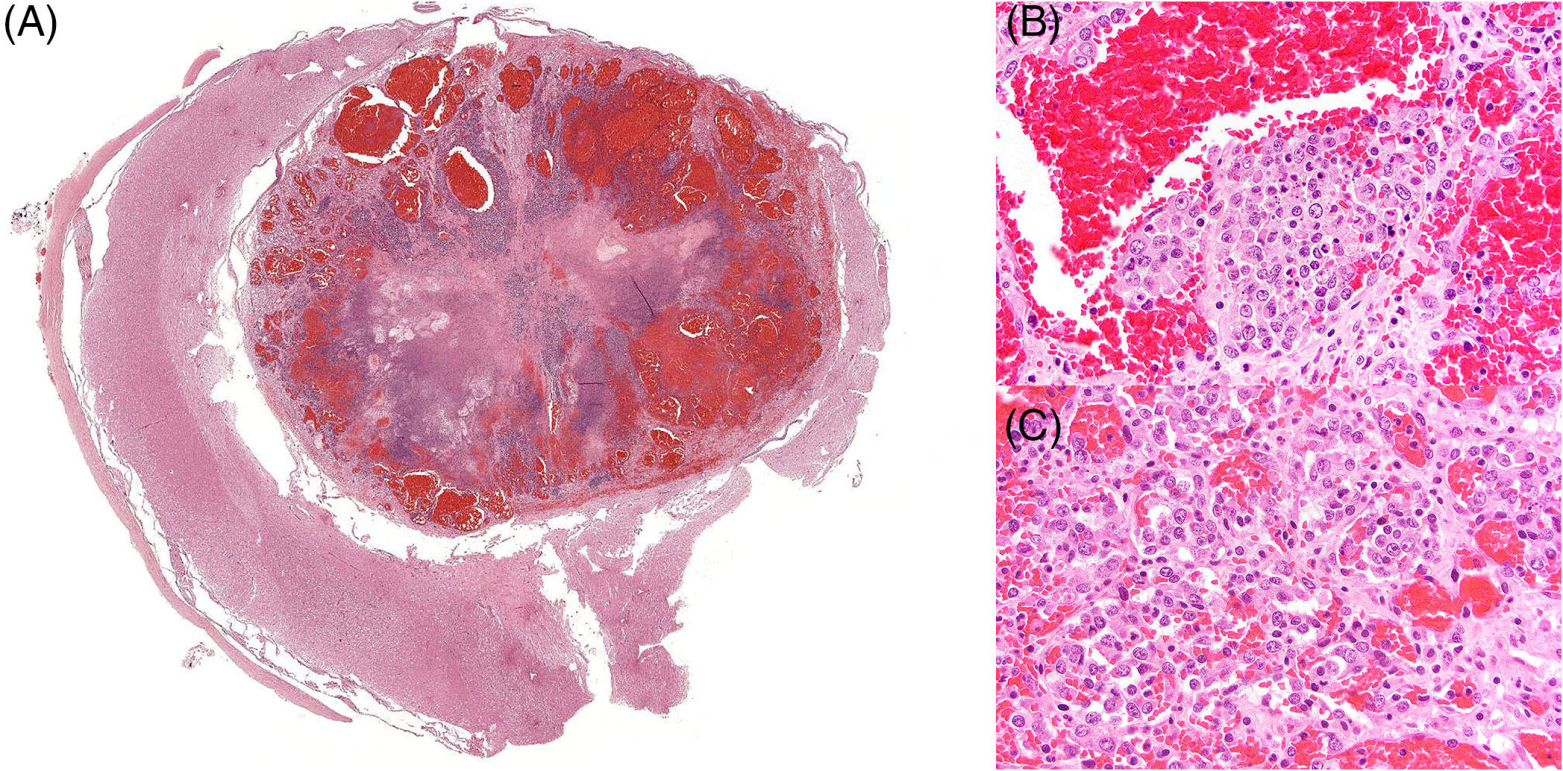
Hematoxylin‐eosin, 2× magnification. (A) A prominently vascular and widely necrotic neoplastic mass arises within and peripheralizes the spinal medulla. The mass consists of unevenly ectatic vascular channels and lacunae delineated by thin fibrous septa. (B) Hematoxylin‐eosin, 20× magnification. Neoplastic endothelial cells are haphazardly stratified and exhibit anisokaryosis and anisocytosis. (C) Hematoxylin‐eosin, 40× magnification. Neoplastic cells form irregular vascular channels lined by multilayered neoplastic endothelial cells that have frequent cytomegaly and karyomegaly.

On necropsy, at the approximate level of the third to fifth cervical spinal segment, was a 0.7 cm × 0.9 cm, well‐delineated, ovoid slate gray to light blue expansile mass that effaced approximately 75% of the neuroparenchyma (Figure 2A,B). Histologically, the mass replaced the right ventral and right dorsal gray matter, compressed the surrounding white matter, and displaced the ventral median fissure laterally (Figure 3A). The periphery of the mass was formed by variably sized vascular channels lined by a single layer of endothelial cells (Figure 3B). These vascular spaces merged with a central core of coagulative necrosis and transitioned into regions that were lined by markedly atypical, neoplastic polygonal to spindled endothelial cells arranged in multiple layers forming irregular clusters and cords (Figure 3C). All neoplastic cells had variable amounts of bland, lightly eosinophilic cytoplasm, with a round to ovoid euchromatic nuclei. The mitotic rate was mild to moderate with up to 10 mitotic figures in 10 400× fields (2.37 mm^2^). The surrounding neuroparenchyma had white matter vacuolation with digestion chambers and scattered spheroids. Other comorbid histologic findings were limited to a moderate fibrinous pleuritis of unknown etiopathogenesis. No evidence of neoplastic disease was present in any other organs examined, either grossly or histologically.

## 4 DISCUSSION

Among proliferative vascular disorders, developmental malformations and benign neoplasms can be diagnostically challenging on light microscopy. According to a recent systematic review of such disorders in the CNS of animals, a range of those lesions may present as a focal intraparenchymal mass, including arteriovenous malformation, cavernous hemangioma, capillary hemangioma, capillary telangiectasis, and venous angioma.^10^ Histologically, the mass encountered in our patient had a unique, divergent histologic pattern. The periphery of the mass, with its variably‐ sized vascular channels and bland endothelial cell lining, was consistent with a cavernous hemangioma, but the degree of atypia, high mitotic rate and extensive coagulative necrosis indicated malignant features, consistent with a final diagnosis of HSA.^10^ Despite the focal presentation of the lesion, the constellation of concurrent benign and malignant histologic features in this case were thus consistent with a cavernous hemangioma that transformed into an HSA. Although such a transformation is sparsely documented in the literature, a large study reviewing 212 dogs with cutaneous hemangioma and HSA does note progression from SC hemangioma to HSA in sun‐exposed skin.^20^ Similar malignant transformation has been reported in humans.^21^ Hemangiosarcoma affecting the CNS traditionally results from metastasis from a distant primary site, and most commonly comprises endothelial cells exhibiting highly atypical, malignant features. True primary HSA of the CNS is not described in the dog, but it is the most common sarcoma metastatic to the CNS.^11^ Based on the absence of a distant primary source at necropsy, and with regard to the focal, solitary nature of the lesion in this dog, with features typical of a hemangioma, the mass was considered to have arisen primarily in the spinal cord.

Compared with extradural and intradural‐extraparenchymal locations, intraparenchymal tumors are the least common spinal neoplasms and make up approximately 16% of spinal tumors.^19^ A previous histopathologic study of 53 dogs with intraparenchymal tumors found that these tumors were more likely to be primary than secondary, and that 100% of dogs with cervical neurolocalization on neurologic examination had primary tumors.^19^ This same report found that the most likely cancers (with equal prevalence) to metastasize to the intraparenchymal location of the spinal cord were HSA and transitional cell carcinoma. Furthermore, a report of MRI features reviewing 21 cases of histopathologically confirmed CNS HSA reported 5 intraparenchymal metastatic lesions in the cervical spinal cord segment.^11^ Accordingly, antemortem assessment regarding primary or secondary disease should rely on staging information, rather than tumor location in the spinal cord.

The mass described here had MRI features consisting of T2 hypointensity relative to muscle, T1 isointensity, strong heterogenous contrast enhancement, T2* susceptibility artifact, and marked regional T2 hyperintensity of the spinal parenchyma without contrast enhancement, consistent with edema. A previous report of MRI features of CNS HSA, including intraparenchymal lesions, similarly identified the presence of T2* susceptibility artifact, associated moderate to severe perilesional edema, and moderate to strong heterogenous or ring‐like contrast enhancement as consistent MRI findings.^11^ T2* susceptibility thus appears to be a preeminent feature of HSA and may help define this neoplasm relative to other lesions. T2* susceptibility most frequently is identified with paramagnetic blood degradation productions in association with hemorrhage. Differential diagnoses therefore for intraparenchymal T2* susceptibility and presumed hemorrhage include bleeding neoplasms (such as HSA), coagulopathy, hematoma, intraparenchymal intervertebral disk extrusion, acute noncompressive nucleus pulposus extrusion, meningomyelitis, hemorrhagic myelomalacia, vascular malformation, parasitic migration, or external or iatrogenic trauma.^22^, ^23^, ^24^, ^25^, ^26^ Location of the lesion relative to the intervertebral disk space, signalment, history, CSF analysis, and coagulation testing will further differentiate among these disorders.

In summary, our report provides the first MRI and histopathologic features of a rare primary intraparenchymal HSA in a German shepherd dog that was presented with cervical myelopathy. Primary HSA should be considered a differential diagnosis in patients with intraparenchymal spinal cord masses that display T2* signal void and contrast enhancement on MRI.

## CONFLICT OF INTEREST DECLARATION

Authors declare no conflict of interest.

## OFF‐LABEL ANTIMICROBIAL DECLARATION

Authors declare no off‐label use of antimicrobials.

## INSTITUTIONAL ANIMAL CARE AND USE COMMITTEE (IACUC) OR OTHER APPROVAL DECLARATION

Authors declare no IACUC or other approval was needed.

## HUMAN ETHICS APPROVAL DECLARATION

Authors declare human ethics approval was not needed for this study.
